# Prediction of 90-day mortality in patients without diabetes by severe hypoglycemia: blood glucose level as a novel marker of severity of underlying disease

**DOI:** 10.1007/s00592-014-0640-9

**Published:** 2014-09-07

**Authors:** Tetsuro Tsujimoto, Ritsuko Yamamoto-Honda, Hiroshi Kajio, Miyako Kishimoto, Hiroshi Noto, Remi Hachiya, Akio Kimura, Masafumi Kakei, Mitsuhiko Noda

**Affiliations:** 1 Department of Diabetes, Endocrinology, and Metabolism, National Center for Global Health and Medicine, Tokyo, Japan; 2 Division of General Medicine, Jichi Medical University Graduate School of Medicine, Tochigi, Japan; 3 Department of Diabetes Research, Diabetes Research Center, National Center for Global Health and Medicine, 1-21-1 Toyama, Shinjuku-ku, Tokyo, 162-8655 Japan; 4 Department of Emergency Medicine and Critical Care, Center Hospital, National Center for Global Health and Medicine, Tokyo, Japan; 5 First Department of Comprehensive Medicine, Saitama Medical Center, Jichi Medical University School of Medicine, Saitama, Japan

**Keywords:** Severe hypoglycemia, Cancer, Liver cirrhosis, Sepsis, Non-diabetes, 90-Day mortality

## Abstract

**Aim:**

The present study examined the clinical conditions and predictors of death in non-diabetic patients with pre-hospital severe hypoglycemia.

**Materials and methods:**

From January 2006 to March 2012, we retrospectively reviewed the medical records to assess the patients with pre-hospital severe hypoglycemia at a national center in Japan. Severe hypoglycemia was defined as the presence of any hypoglycemic symptoms requiring the medical assistance of another person. The patients were followed up for 90 days after the severe hypoglycemia episode.

**Results:**

A total of 59,602 consecutive cases that visited the emergency room were screened, and 530 patients with severe hypoglycemia were included in the subsequent analysis. The mean blood glucose levels in the non-diabetes (non-DM, *n* = 163) and diabetes (DM, *n* = 367) groups were 42.9 and 33.7 mg/dL, respectively. The incidence of extremely abnormal QT prolongation (corrected QT interval ≥0.50 s) was high in both groups [22.1 vs. 14.7 % (*P* = 0.11)]. Mortalities within 90 days after severe hypoglycemia were significantly higher in the non-DM group than in the DM group [20.3 vs. 1.6 % (*P* < 0.001)]. In addition to patient age, preexisting advanced liver disease and cancer, and the coexistence of sepsis, a blood glucose level of <40 mg/dL was identified as a strong predictor of death in the non-DM group using multivariate Cox proportional hazards models (hazard ratio 3.75; 95 % confidence interval 1.52–9.27; *P* = 0.004).

**Conclusions:**

Death in non-diabetic patients with severe hypoglycemia was independently associated not only with age, advanced liver disease, cancer, and sepsis, but also with the blood glucose level upon arrival.

## Introduction

Severe hypoglycemia is an extremely dangerous event [1, 2]. Several studies have suggested that hypoglycemia in patients with diabetes is associated with increased cardiovascular events and death [3–5]. Moreover, some reports have indicated that hypoglycemia might also be associated with a higher mortality in patients with critical illness [6–8]. However, whether iatrogenic hypoglycemia caused by glucose-lowering agents is associated with increased mortality or whether the underlying diseases causing the hypoglycemia are associated with the high mortality has been unclear and controversial.

Although hypoglycemia in patients with diabetes is mostly iatrogenic and is mainly caused by glucose-lowering agents, such as insulin and sulfonylureas, hypoglycemia in patients without diabetes can be caused by many factors including malnutrition, alcohol abuse, hepatic failure, and sepsis [9]. A recent study has suggested that severe hypoglycemia in critically ill patients who are not receiving insulin treatment might be associated with increased mortality [8], and another study has shown that hypoglycemia at the time of hospital admission was associated with in-hospital mortality in patients with sepsis [10]. However, a systematic study investigating predictors of death in non-diabetic patients with non-iatrogenic hypoglycemia has not been previously performed. The present study examined the clinical conditions and predictors of death in non-diabetic patients with pre-hospital severe hypoglycemia.

## Methods

### Study design

We conducted a retrospective cohort study of non-diabetic patients who had been transported by ambulance and were diagnosed as having severe hypoglycemia at the National Center for Global Health and Medicine Hospital in Tokyo, Japan, between January 1, 2006, and March 31, 2012. Severe hypoglycemia was defined as the presence of any hypoglycemic symptoms that could not be resolved by the patients themselves and that required the medical assistance of another person after visiting the emergency room by ambulance [11]. Blood glucose levels were mainly measured at a central laboratory (78 %, 411/529), although some were measured using a blood glucose meter (22 %, 118/529). All blood glucose levels of <20 mg/dL were checked at a central laboratory. In one patient, the blood glucose level was not measured prior to treatment. We preferentially referred to the blood glucose data measured at a central laboratory. We assessed the patients’ characteristics, general conditions, and electrocardiograms upon arrival, as well as any complications with severe hypoglycemia and the clinical outcomes. At least two specialists in both diabetology and internal medicine independently reviewed all the data, including the clinical records, laboratory data, and electrocardiograms. Disagreements between the reviewers were resolved by a third internist. Diabetes was confirmed when the patient had been previously diagnosed as having diabetes or was being treated with antidiabetic medicines, and non-diabetic patients were defined as those without diabetes. Patients with cardiopulmonary arrest upon arrival were excluded from this study. We analyzed the data using only the latest hospital visit for each individual. All the eligible patients in this study were followed up for 90 days after severe hypoglycemia. This study was approved by the institutional review board of the National Center for Global Health and Medicine Hospital.

### Clinical conditions

We assessed the consciousness level and the vital signs of all the study patients. The consciousness level during severe hypoglycemia was evaluated using the Glasgow Coma Scale (GCS) score [12]. The GCS score is composed of three parameters: best eye response between 1 and 4, best verbal response between 1 and 5, and best motor response between 1 and 6. The GCS score can range from 3 to 15, with 3 being the worst possible score and 15 being the best possible score. The body temperature upon arrival was measured at the rectum, axilla, and/or tympanic membrane, and we preferentially referred to the rectal temperature. Mild hypothermia was defined as a body temperature <35 °C, and moderate to severe hypothermia was defined as <32 °C [13]. Blood pressure was measured upon arrival. Severe hypertension was defined as a systolic blood pressure ≥180 mmHg and/or a diastolic blood pressure ≥120 mmHg [14]. Newly diagnosed diseases during episodes of severe hypoglycemia were assessed using the medical records, laboratory data, electrocardiograms, and radiological images. Cardiovascular events were defined as coronary heart disease requiring treatment with revascularization or stroke confirmed by radiological images. Sepsis was defined as systemic inflammatory response syndrome in response to infection. Systemic inflammatory response syndrome was regarded as the presence of two or more of the following criteria: body temperature >38 or <36 °C, heart rate >90 beats per minute, respiratory rate >20 breaths per minute, and white blood cell >12,000 per mm^3^. The corrected QT interval (QTc) was calculated using Bazett’s formula: QTc = QT interval ÷ square root of the RR interval. A QTc ≥0.44 s was considered to indicate abnormal prolongation, and those ≥0.50 s were considered to be highly abnormal [15, 16].

### Statistical methods

Patients were initially categorized into the diabetes (DM) or non-diabetes (non-DM) group. Data were presented as the number (%), the mean with standard deviation (SD), or the median with the lower and upper ends of the interquartile range (IQR). Continuous variables were compared using *t* tests or Wilcoxon rank-sum tests. Categorical variables were compared using chi-squared tests or Fisher’s exact tests. To analyze the GCS score, body temperature, and blood pressure upon arrival, the subjects were divided into two groups according to a cut-off blood glucose level of 40 mg/dL (to convert blood glucose to mmol/L, multiply by 0.0555), which approximated the overall mean value. Cox proportional hazards models were used to identify risk factors associated with death in non-diabetic patients with severe hypoglycemia. The candidate variables included in this model were age, sex, preexisting diseases, QT prolongation, coexisting sepsis, and blood glucose levels. We performed univariate analyses to determine the magnitude of unadjusted associations and subsequently performed multivariate analyses using the variables that were deemed significant in the univariate analyses. Kaplan–Meier analyses were used to assess mortality within 90 days after severe hypoglycemic events, and groups were compared using the log-rank test. *P* values <0.05 according to a two-sided test were considered statistically significant for all the tests. All the analyses were performed using Stata software, version 11.1 (Stata Corp, College Station, TX, USA).

## Results

**Fig. 1:**
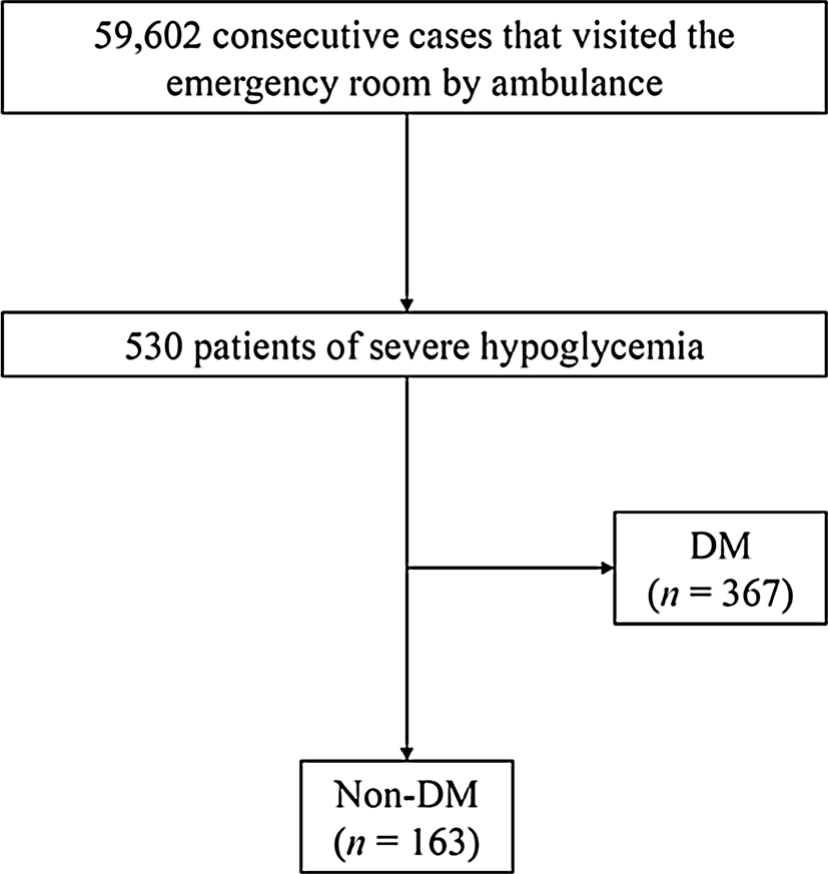
Flowchart of study participants. *Non-DM* non-diabetes, *DM* diabetes mellitus

A total of 59,602 consecutive cases that visited the emergency room by ambulance were screened, and 530 patients with severe hypoglycemia met the criteria for inclusion in this study (Fig. 1). The clinical characteristics of this study population upon arrival are presented in Table 1. In the DM group, the numbers (%) of type 1 diabetes, type 2 diabetes, and other diabetes were 63 (17.2 %), 293 (79.8 %), and 11 (3.0 %), respectively. In the non-DM (*n* = 163) and DM (*n* = 367) groups, the mean ± SD blood glucose levels were 42.9 ± 23.2 and 33.7 ± 15.7 mg/dL, respectively (*P* < 0.001). Patient age and sex were not significantly different between the non-DM and DM groups. The prevalence of known cardiovascular disease and preexisting hypertension in the DM group was significantly higher, and the estimated GFR in the DM group was significantly lower than that in the non-DM group. The patients in the non-DM group had a broad range of causes of severe hypoglycemia, with the major ones being malnutrition, alcohol abuse, post-gastrectomy, and infection. Meanwhile, almost all the patients of severe hypoglycemia in the DM group were caused by glucose-lowering medications, and the causes of severe hypoglycemia were completely different between the non-DM and DM groups.

**Table 1:** Clinical profiles upon arrival

Characteristics	Non-DM (*n* = 163)	DM (*n* = 367)	*P* value
Age (years)	63.3 ± 20.0	66.1 ± 16.5	0.08
Women	58 (35.6 %)/163	120 (32.7 %)/367	0.51
History of cardiovascular disease	18 (11.0 %)/163	69 (18.8 %)/367	0.02
Preexisting disease			
Hypertension	31 (19.0 %)/163	223 (60.8 %)/367	<0.001
Atrial fibrillation	5 (3.1 %)/163	19 (5.2 %)/367	0.36
Advanced liver disease	7 (4.3 %)/163	15 (4.1 %)/367	>0.99
Cancer other than HCC	11 (6.8 %)/163	11 (3.0 %)/367	0.05
Blood glucose (mg/dL) (*n* = 529)	42.9 ± 23.2	33.7 ± 15.7	<0.001
Creatinine (mg/dL) (*n* = 490)	1.06 ± 0.83	1.36 ± 1.45	0.01
Estimated GFR (mL/min/1.73 m^2^) (*n* = 490)	72.6 ± 37.3	61.6 ± 32.2	<0.001
Causes of severe hypoglycemia			
Glucose-lowering medications	3 (1.8 %)/163^a^	342 (93.2 %)/367	<0.001
Malnutrition	65 (39.9 %)/163	4 (1.1 %)/367	<0.001
Alcohol	38 (23.3 %)/163	10 (2.7 %)/367	<0.001
Post-gastrectomy	13 (8.0 %)/163	4 (1.1 %)/367	<0.001
Infection	14 (8.6 %)/163	4 (1.1 %)/367	<0.001
Others	30 (18.4 %)/163	3 (0.8 %)/367	<0.001

Data are represented as the number, number (%)/total number, or mean ± SD. ***N*** *on-DM* non-diabetes, *DM* diabetes mellitus, *HCC* hepatocellular carcinoma, *GFR* glomerular filtration rate

Infection was defined as the presence of a bacterial or viral infectious disease

SI conversion factors: to convert blood glucose to mmol/L, multiply by 0.0555; to convert creatinine to μmol/L, multiply by 88.4

History of cardiovascular disease was defined as a history of myocardial infarction, angina pectoris, stroke, or peripheral artery disease

Advanced liver disease was defined as the presence of cirrhosis or hepatocellular carcinoma

Cancer was defined as any cancer excluding fully healed cancer

Estimated GFR was calculated using the following formula: estimated GFR (mL/min/1.73 m^2^) = 194 × Cre^−1.094^ × age^−0.287^ (×0.739, if the patient was female)

^a^Two patients attempted suicide using insulin, and one patient took sulfonylurea incorrectly. All three patients were safely discharged from the hospital

The clinical conditions and events upon arrival and the mortality in patients with severe hypoglycemia are shown in Table 2. The GCS scores in the non-DM and DM groups were 10.7 ± 4.2 and 10.0 ± 4.1, respectively, and these scores were not significantly different. In each group, the GCS scores of the patients with a blood glucose level <40 mg/dL were significantly lower than those with a blood glucose level ≥40 mg/dL [8.4 ± 4.4 vs. 13.0 ± 2.5 in the non-DM group (*P* < 0.001); 9.0 ± 3.9 vs. 13.0 ± 3.0 in the DM group (*P* < 0.001)]. The systolic and diastolic blood pressures were significantly lower in the non-DM group than in the DM group. The incidence of severe hypertension in the DM group was three times as high as that in the non-DM group. The body temperature was significantly lower in the non-DM group than that in the DM group, and the incidence of moderate to severe hypothermia was significantly higher in the non-DM group than in the DM group. The body temperature was significantly lower in patients with a blood glucose level <40 mg/dL, compared with those with a blood glucose level ≥40 mg/dL [34.5 ± 2.7 vs. 35.5 ± 2.2 °C in the non-DM group (*P* = 0.01); 35.4 ± 1.0 vs. 36.0 ± 1.0 °C in the DM group (*P* < 0.001)]. The serum potassium levels were significantly lower in the DM group than in the non-DM group. The incidence of QT prolongation was high in both groups. Although a QTc ≥0.50 s in the non-DM group was not associated with the blood glucose level and potassium level upon arrival, the body temperature was significantly lower in patients with a QTc ≥0.50 s, compared with those with a QTc <0.50 s [32.8 ± 3.2 vs. 35.7 ± 1.5 °C (*P* < 0.001)]. Although no significant difference was seen between the groups, the incidence of new-onset cardiovascular disease during an episode of severe hypoglycemia was higher in the DM group than in the non-DM group. The patients with new-onset atrial fibrillation in the non-DM group had a significantly higher incidence of a QTc ≥0.50 s than those without new-onset atrial fibrillation (*P* = 0.04). Fatal arrhythmias upon arrival, such as complete atrioventricular block, ventricular tachycardia, ventricular fibrillation, and torsade de pointes, were not observed in the study patients. The hospitalization rates in the non-DM and DM groups were 54.6 and 27.8 %, respectively, and these were significantly different (*P* ≤ 0.001). The lengths of hospital stay in both groups were not significantly different between patients with a blood glucose level of ≤40 and ≥40 mg/dL [8 (3–21) days vs. 7 (2–14) days in the non-DM group (*P* = 0.35); 8 (2–19) days vs. 9 (2–20) days in the DM group (*P* = 0.97)]. In the non-DM group, the mortality rate was 20.3 %, which was more than ten times as high as that in the DM group. The major causes of death in the non-DM group were infection (51.5 %), advanced liver disease (15.2 %), and cancer (9.1 %), while those in the DM group were infection (83.3 %) and advanced liver disease (16.7 %).

**Table 2:** Clinical conditions and events upon arrival and mortality in patients with severe hypoglycemia

Conditions, events, and mortality	Non-DM	DM	*P* value
GCS score (*n* = 518)	10.7 ± 4.2	10.0 ± 4.1	0.07
Systolic blood pressure (mmHg) (*n* = 521)	126.0 ± 31.1	165.6 ± 35.4	<0.001
Diastolic blood pressure (mmHg) (*n* = 515)	73.2 ± 22.5	80.2 ± 22.0	<0.001
Severe hypertension	13 (8.1 %)/160	122 (33.6 %)/363	<0.001
Preexisting hypertension (+)	3 (9.7 %)/31	87 (39.0 %)/223	0.001
Preexisting hypertension (−)	10 (7.8 %)/129	35 (25.0 %)/140	<0.001
Body temperature (*n* = 477)	35.0 ± 2.5	35.6 ± 1.0	<0.001
Mild hypothermia	45 (29.8 %)/151	72 (22.0 %)/328	0.06
Moderate or severe hypothermia	18 (11.9 %)/151	1 (0.3 %)/328	<0.001
Serum potassium (mEq/L) (*n* = 503)	3.9 ± 0.8	3.7 ± 0.7	<0.001
<3.5 mEq/L	42 (26.6 %)/158	126 (35.7 %)/353	0.04
QT prolongation (*n* = 95)			
QTc ≥0.44 s	54 (56.8 %)/95	108 (58.7 %)/184	0.76
QTc ≥0.50 s	21 (22.1 %)/95	27 (14.7 %)/184	0.11
Newly diagnosed complications upon arrival			
Cardiovascular disease	1 (0.6 %)/163	5 (1.36 %)/367	0.67
Atrial fibrillation	7 (4.3 %)/163	14 (3.8 %)/367	0.81
Coexisting sepsis	29 (17.8 %)/163	15 (4.1 %)/367	<0.001
Death within 90 days after severe hypoglycemia	33 (20.3 %)/163	6 (1.6 %)/367	<0.001

Data are represented as the mean ± SD or number (%)/total number. *GCS* Glasgow Coma Scale, *DM* diabetes mellitus, *QTc* corrected QT interval calculated using Bazett’s formula

Severe hypertension was defined as systolic blood pressure ≥180 mmHg and/or diastolic blood pressure ≥120 mmHg

Preexisting hypertension was confirmed when the patient was being treated with antihypertensive medication or had been previously diagnosed as having hypertension

Mild hypothermia was defined as a body temperature <35 °C, and moderate to severe hypothermia was defined as <32 °C

Serum potassium levels were measured upon arrival

QTc was calculated using Bazett’s formula: QTc = QT interval ÷ square root of the RR interval

Cardiovascular disease was defined as coronary heart disease requiring treatment with revascularization or stroke that was confirmed on radiological images

Sepsis was defined as systemic inflammatory response syndrome in response to infection. Systemic inflammatory response syndrome was regarded as the presence of two or more of the following criteria: body temperature >38 or <36 °C, heart rate >90 beats per minute, respiratory rate >20 breaths per minute, and white blood cell >12,000 per mm^3^

**Fig. 2:**
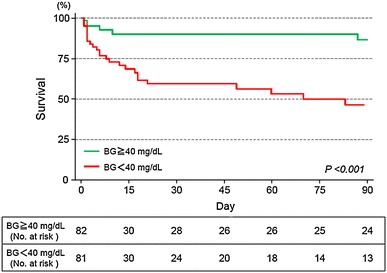
Kaplan–Meier analysis of the time to death after severe hypoglycemia. *BG* blood glucose

Analyses of the clinical variables in patients with and those without death in the non-DM group are shown in Table 3. Age, preexisting advanced liver disease and cancer, coexisting sepsis, and the blood glucose level differed significantly between the two groups when examined using univariate analyses. When a Cox proportional hazards regression was used, these five variables were independently associated with death, and a blood glucose level of <40 mg/dL was one of the strongest predictors (hazard ratio 3.75; 95 % confidence interval 1.52–9.27; *P* = 0.004). Kaplan–Meier survival curves for the patients with blood glucose levels of <40 and ≥40 mg/dL in the non-DM group are shown in Fig. 2. Even though causes of severe hypoglycemia such as malnutrition, alcohol, and post-gastrectomy were included in the multivariate analysis, only these five variables were independently associated with death.

**Table 3:** Analyses of clinical variables according to death or survival in non-diabetic patients with severe hypoglycemia

Variable	Hazard ratio	95 % CI	*P* value
Univariate analysis			
Age (years)	1.03	1.01–1.06	0.003
≥65	2.64	1.14–6.10	0.02
Women	0.81	0.40–1.63	0.56
Preexisting diseases			
Hypertension	1.58	0.75–3.32	0.22
Atrial fibrillation	1.44	0.34–6.05	0.61
Advanced liver disease	7.40	2.79–19.63	<0.001
Cancer	2.90	1.01–8.32	0.04
QTc ≥0.50 s	1.26	0.33–4.80	0.72
Coexisting sepsis	3.91	1.96–7.82	<0.001
Blood glucose (mg/dL)	0.96	0.94–0.98	<0.001
<40	4.65	1.91–11.31	0.001
Multivariate analysis			
Age (years) ≥65	3.42	1.33–8.81	0.01
Advanced liver disease	9.68	3.24–28.86	<0.001
Cancer	3.64	1.22–10.88	0.02
Sepsis	3.17	1.55–6.48	0.002
Blood glucose (mg/dL) <40	3.75	1.52–9.27	0.004

Data are represented as the hazard ratio or 95 % CI. To convert blood glucose to mmol/L, multiply by 0.0555

Advanced liver disease was defined as the presence of cirrhosis or hepatocellular carcinoma

Cancer was defined as any cancer excluding hepatocellular carcinoma and fully healed cancer

QTc was calculated using Bazett’s formula: QTc = QT interval ÷ square root of the RR interval

Sepsis was defined as the presence of systemic inflammatory response syndrome in response to infection. Systemic inflammatory response syndrome was regarded as the presence of two or more of the following criteria: body temperature >38 or <36 °C, heart rate >90 beats per minute, respiratory rate >20 breaths per minute, and white blood cell >12,000 per mm^3^

*CI* confidence interval

## Discussion

This systematic study is, to the best of our knowledge, the first to report that death in non-diabetic patients with pre-hospital severe hypoglycemia was independently associated not only with age, advanced liver disease, cancer, and sepsis, but also with the blood glucose level upon arrival.

Hypoglycemia leads to the activation of the sympathoadrenal system, and the release of epinephrine and norepinephrine results in hemodynamic changes [2, 17, 18]. Moreover, profound hypoglycemia causes neuroglycopenic symptoms, including seizure and coma. Many patients with severe hypoglycemia exhibited low consciousness levels, regardless of the presence or absence of diabetes, and the consciousness levels were significantly lower in patients with lower blood glucose levels. Blood pressure during severe hypoglycemia was significantly higher in patients with diabetes than in those without diabetes. Non-diabetic patients with severe hypoglycemia had a broad range of causes for their conditions, such as malnutrition and alcohol abuse, and their illnesses might have led to an insufficient elevation in blood pressure. In addition, previous studies have shown that hypothermia was associated with hypoglycemia [2, 19]. However, the body temperature of patients with severe hypoglycemia, particularly of non-diabetic patients with severe hypoglycemia, has rarely been examined in clinical settings. This study suggested that mild to severe hypothermia during an episode of severe hypoglycemia was often observed in patients with or without diabetes, and hypothermia was more frequent in patients with lower blood glucose levels than in those with higher blood glucose levels. Although the coexistence of sepsis could lead to hypothermia, thereby influencing the body temperature, intracellular glycopenia at the thermoregulatory center in the hypothalamus might also be one of the central causes of hypothermia [19]. Because hypothermia can lead to further serious outcomes, including lethal arrhythmias, the association between hypoglycemia and arrhythmias should be investigated. The serum potassium levels in patients with severe hypoglycemia have never been sufficiently clarified. Several factors including blood pH, insulin and catecholamine levels, potassium intake, and renal function might influence the serum potassium levels in patients with severe hypoglycemia. Similar to hypothermia, hypokalemia also increases the risk of lethal arrhythmias.

Several studies have reported an association between hypoglycemia and QT prolongation in patients with diabetes [2, 20–22]. This study demonstrated that not only diabetic patients, but also non-diabetic patients with severe hypoglycemia frequently had an abnormal QT prolongation. Although many causes could lead to acquired long QT syndrome, hypothermia in patients with severe hypoglycemia might be strongly associated with prolonged QT intervals. Because patients with QT prolongation might have torsade de pointes and might have a higher mortality than those without QT prolongation [15, 23, 24], QT prolongation could be another threat in patients with severe hypoglycemia. In addition, although the association between highly abnormal QT prolongation and new-onset atrial fibrillation in non-diabetic patients with severe hypoglycemia may be supported by a recent study that revealed an association between QT prolongation and the onset of atrial fibrillation [25], further research is required to confirm an association.

Previous reports have suggested that severe hypoglycemia is associated with increased mortality [6–8]. However, little data are available regarding the outcomes of patients with severe and non-iatrogenic hypoglycemia. Our study showed that non-diabetic patients with severe hypoglycemia frequently died in the absence of glucose-lowering interventions. The characteristics of non-diabetic patients with severe hypoglycemia differed substantially from those of diabetic patients, which might have influenced the patient outcomes. In addition to age, advanced liver disease, cancer, and sepsis, the blood glucose level was independently associated with death in non-diabetic patients with severe hypoglycemia. Severe hypoglycemia in elderly and cancer patients might be one of the important findings for their critical condition. When patients with liver cirrhosis and/or hepatocellular carcinoma develop non-iatrogenic hypoglycemia, gluconeogenesis and glycogenolysis might be severely impaired—suggesting extremely serious liver damage. Meanwhile, hypoglycemia is sometimes complicated by the presence of sepsis [26]. Although sepsis-induced hypoglycemia might arise from a decrease in glucose production and an increase in glucose uptake by macrophage-rich tissues [27, 28], the causes remained unclear. However, sepsis with hypoglycemia could be much more serious than those without hypoglycemia [10]. Additionally, blood glucose is a major energy source in almost all organs and is essential for survival. When blood glucose levels are low in patients with non-iatrogenic hypoglycemia, insulin secretion is promptly suppressed and counter-regulatory hormones, such as glucagon, catecholamines, cortisol, and growth hormone, are secreted in considerable quantities, elevating the blood glucose level. However, when these biological reactions do not lead to an increase in the blood glucose level, the underlying diseases might be extremely severe, and all the organs are likely facing critical situations as a result of the underlying diseases and the hypoglycemic conditions. Although unknown variables might exist, the blood glucose level during episodes of severe hypoglycemia might reflect the severity of the underlying disease. Moreover, non-diabetic patients might not adapt adequately to hypoglycemia compared with diabetic patients. This might be one of the reasons for the higher mortality of patients without diabetes than that of patients with diabetes.

The present study had several limitations. First, missing data [including acute physiology and chronic health evaluation (APACHE) II scores] and missing confounding factors might have influenced the results. However, there is no systematic study investigating the detailed conditions and predictors of death in non-diabetic patients with pre-hospital severe hypoglycemia. Therefore, we believe that the present study supplies very important information about severe hypoglycemia in clinical settings. Second, this study was performed at a single national center, and the study patients were from a specific geographical area. Further research at multiple centers throughout the world is needed. Third, patients with pre-hospital cardiopulmonary arrest could not be examined. Some patients with severe hypoglycemia might have died from cardiovascular events, lethal arrhythmias, or other critical events in pre-hospital settings. Nevertheless, we believe that our study provides novel information about severe hypoglycemia.

## Conclusion

The present study revealed that in addition to patient age, preexisting advanced liver disease, cancer, and coexisting sepsis, the blood glucose level was one of the strongest predictors of death after an episode of severe hypoglycemia in non-diabetic patients. Mortality of non-diabetic patients with pre-hospital severe hypoglycemia was extremely high, and more attention should be paid to their blood glucose levels.
